# A compilation of research working groups on drug utilisation across Europe

**DOI:** 10.1186/1756-0500-7-143

**Published:** 2014-03-13

**Authors:** Mònica Sabaté, Juan Fernando Pacheco, Elena Ballarín, Pili Ferrer, Hans Petri, Joerg Hasford, Marieke Wilma Schoonen, Marietta Rottenkolber, Joan Fortuny, Joan-Ramon Laporte, Luisa Ibáñez

**Affiliations:** 1 Fundació Institut Català de Farmacologia, Pg. Valld’Hebron 119-129, Barcelona 08035, Spain; 2 Servei de Farmacologia Clínica, Hospital Universitari Vall d’Hebron, Barcelona, Spain; 3 Departament de Farmacologia, Terapèutica i Toxicologia, Universitat Autònoma de Barcelona, Pg. Vall d’Hebron 119-129, Barcelona 08035, Spain; 4 Hoffmann- La Roche, 6 Falcon Way, Shire Park, Welwyn Garden City AL7 1TWLondon, UK; 5 Institute for Medical Information Sciences, Biometry and Epidemiology Ludwig Maximillians Universität-München, Marchioninistr. 15, 81377 Munich, Germany; 6 Center for Observational Research, Amgen, ltd., 1, Uxbridge Business Park, Sanderson Road, Uxbridge, Middlesex, UK; 7 Global Clinical Epidemiology Department, Novartis Pharma AG, Gran Vía Corts Catalanes, 764, 08013 Barcelona, Spain

**Keywords:** Pharmacoepidemiology, Pharmacovigilance, European network, Drug utilisation, Drug consumption, National databases, Review

## Abstract

**Background:**

The assessment of the benefit-risk of medicines needs careful consideration concerning their patterns of utilization. Systems for the monitoring of medicines consumption have been established in many European countries, and several international groups have identified and described them. No other compilation of European working groups has been published.

As part of the PROTECT project, as a first step in searching for European data sources on the consumption of five selected groups of medicines, we aimed to identify and describe the main characteristics of the existing collaborative European working groups.

**Findings:**

Google and bibliographic searches (PubMed) of articles containing information on databases and other sources of drug consumption data were conducted. For each working group the main characteristics were recorded.

Nineteen selected groups were identified, focusing on: a) general drug utilisation (DU) research (EuroDURG, CNC, ISPE’S SIG-DUR, EURO-MED-STAT, PIPERSKA Group, NorPEN, ENCePP, DURQUIM), b) specific DU research: b.1) antimicrobial drugs (ARPAC, ESAC, ARPEC, ESGAP, HAPPY AUDIT), b.2) cardiovascular disease (ARITMO, EUROASPIRE), b.3) paediatrics (TEDDY), and b.4) mental health/central nervous system effects (ESEMeD, DRUID, TUPP/EUPoMMe). Information on their aims, methods and activities is presented.

**Conclusions:**

We assembled and updated information on European working groups in DU research and in the utilisation of five selected groups of drugs for the PROTECT project. This information should be useful for academic researchers, regulatory and health authorities, and pharmaceutical companies conducting and interpreting post-authorisation and safety studies. European health authorities should encourage national research and collaborations in this important field for public health.

## Findings

The assessment of the benefit-risk of medicines needs careful consideration concerning their patterns of utilization.

Systems for the monitoring of medicines consumption and assessing their benefit-risk have been established in many European countries. Several international groups have identified and described those systems.

To identify and describe the main characteristics of the existing collaborative European working groups, we conducted a Google and bibliographic searches (PubMed) of articles containing information on databases and other sources of drug consumption data of medicines.

Nineteen selected groups were identified, focusing on: a) general drug utilisation (DU) research (EuroDURG, CNC, ISPE’S SIG-DUR, EURO-MED-STAT, PIPERSKA Group, NorPEN, ENCePP, DURQUIM), b) specific DU research: b.1) antimicrobial drugs (ARPAC, ESAC, ARPEC, ESGAP, HAPPY AUDIT), b.2) cardiovascular disease (ARITMO, EUROASPIRE), b.3) paediatrics (TEDDY), and b.4) mental health/central nervous system effects (ESEMeD, DRUID, TUPP/EUPoMMe). Information on their aims, methods and activities is presented.

## Introduction

Drug utilisation research is defined by the World Health Organization (WHO) as “the development, regulation, marketing, distribution, prescription, dispensing and use of medicines within a society, with special emphasis on the medical, social and economic consequences” [1]. A broad definition should also include the qualitative studies for assessing the appropriateness of drug utilisation and the intervention studies [2]. Drug utilisation research plays a key role in understanding the use of medicines and evaluating the effect of interventions (such as policy changes, reimbursement policy and regulatory decisions) on drug use, thereby ameliorating the quality of care and improving public health.

The Pharmacoepidemiological Research on Outcomes of Therapeutics by a European ConsorTium (PROTECT) study is a collaborative European project which aims to enhance the monitoring of the safety of medicinal products [3]. One of the specific objectives of PROTECT is to build and update an inventory of data sources on the consumption of medicines in the European Union as a tool to estimate the public health impact of several adverse drug events.

Efforts to collect information about the use of medicines in European countries date from the seventies. There is a wide international variability in drug utilisation documented in a WHO-Regional Office for Europe sponsored meeting [4]. In addition, information about the overall use of medicines across European countries is of interest to estimate the public health impact of adverse effects associated with the use of medicines [5,6]. As a first step in searching for European data sources for medicines consumption, we aimed to describe and update the main characteristics of the collaborative international European working groups, networks, and research projects related to drug utilisation.

## Methods

### Search strategy

1. Internet search. The goal was to find institutions, networks and research projects related to drug utilisation in Europe in general and those focused on six groups of drugs, namely: 1) inhaled beta-2 agonists; 2) antibiotics; 3) antidepressants 4) benzodiazepines; 5) anticonvulsants, and 6) calcium channel blockers [3].

2. Bibliographic search: (1990-2010) in PubMed and SIETES (Sistema de Información Esencial en Terapéutica y Salud (http://www.sietes.org), an electronic drug information system in Spanish). Keywords: “databases*”, “drug utilization”, “d*rug utilization research”, “Europe”, “international cooperation”, “international group”, “national databases”, “network”, “pharmacoepidemiology” and “working group”.

### Group selection criteria

Working groups were included if they were European groups, focused on drug utilisation and/or if they were involved in research on the medicines of interest for the PROTECT project. This selection was done according to pre-defined criteria such as regulatory and public health impact, and the potential to investigate a broad range of methodological issues [7].

Groups studying a single condition and/or those focusing only on drugs of no interest for the PROTECT project, or based on a single European country, or not active at the time of search, were excluded.

### Data abstraction

The characteristics of each working group were collected from their websites or from methods and acknowledgement sections in published papers.

These data were analysed in a descriptive manner.

## Results

Twenty-four European working groups on drug utilisation were identified, nineteen of which fulfilled our eligibility criteria. Additional information on excluded groups can be found on (http://www.icf.uab.es/EuropeanWG).

The characteristics of the working groups are described in the Additional file 1: Table S1. Eight groups focused on promoting general drug utilisation research and eleven focused on specific fields.

## Discussion

As far as we know, no other compilation of European collaborative working groups and their sources of data on medicines utilisation have been published.

Nineteen European groups were selected: eight groups were interested in general drug utilisation research or pharmacoepidemiology, with the common objective of compiling information on data sources, either at a European level (e.g., ENCePP in Europe) or at a more restricted geographical level (e.g., NorPen in the Nordic countries). The remaining eleven groups focused their research on specific fields, mainly antimicrobials, cardiovascular conditions, paediatrics, and mental health (e.g., ESAC: antimicrobials, EUROASPIRE: cardiovascular conditions).

Among the groups focusing on general drug utilisation the EURO-MED-STAT widened the initial EuroMedicines project and developed a European database of licensed medicines and their prices in twenty European countries [8].

The CNC project offered a wide range of sources of medicines consumption data for eighteen countries. However, the information is not published but available on a website [9], and it is not clear whether the information has been kept updated.

EnCePP, led by the European Medicines Agency (EMA), was established to strengthen the postmarketing monitoring of medicines. Its website contains a voluntary register of the healthcare databases existing in all European countries including those monitoring drug consumption.

Our search identified several European working groups collecting information on the utilisation of the selected groups of medicine even though their main objective goes beyond the collection of drug consumption data.

As expected, we found wide heterogeneity in the nature and quality of the drug utilisation data among the groups. The main factors determining their heterogeneity were: a) variability in the population coverage, and in the number of countries involved in each working group; b) differences in medicines coding systems, and c) source of the drug utilisation data (e.g., questionnaires to individuals, samples or registers of prescribed/dispensed medicines). In addition, none of the groups, except ESAC, has tried to validate drug utilisation data [10].

Inhaled beta-2 agonists and anticonvulsants did not appear in our search in relation to medicines consumption. This could be explained by the fact that the groups working in those areas concentrate more on risk factors associated with diseases rather than on exposure to medicines.

Most of the initiatives in this field have received public funding. As a consequence a lot of good initiatives and efforts could have been lost if this funding had ended. Funding is decisive in keeping these research working groups ongoing.

The general working groups contribute to the collaboration in pharmacoepidemiology and specifically in drug utilisation research studies across the European countries through improving the research in this field and the quality of health care management. For example, PIPERSKA contributes to enhancing the rational use of drugs in different countries and the Nordic network NorPEN facilitates and promotes safer and more efficient medicines in a public health perspective in the Nordic countries. Among its goals there is the objective to increase quality of research and methodological development within pharmacoepidemiology in the Nordic countries.

The importance and impact of the specific drug utilisation groups in the medical community stems from the collaborative research in drug utilisation and methodological innovation as well as from aspects derived from their field of interest. For instance, the groups focusing on antibiotics such as ESAC, have contributed not only to a specific field such as the use of antibiotics in Europe but to guidelines for methods in drug utilisation research (i.e, study design and comparison of drug utilisation data across different countries). The field of antimicrobial drugs has traditionally interested European researchers due to its relationship with the appearance of resistances. Those groups interested in cardiovascular diseases such as ARITMO or EUROASPIRE work on diseases with a high public health impact [11], and they have been active for a long time. Their importance also lies in their interest in adverse drug reactions and in prognostic factors for disease prevention. Other initiatives such as TEDDY focus on the paediatric population. TEDDY has the purpose of promoting the availability of safe and effective paediatric drugs to overcome the difficulties and complexity of research in this population. It continues the activities through the participation in other on-going projects (ex Global Research in Paediatrics (GRIP)). Finally the research groups focusing on mental diseases derive their importance from the high prevalence of mental diseases worldwide [12].

The strengths of our compilation are the standardised search and the categorising of the information as general and specific-related drug utilisation areas in Europe. The broad search lets us find out about the general groups on drug utilisation as well as those focusing on the selected PROTECT drugs. This paper highlights some of the benefits of international collaboration such as the possibility of sharing and transferring knowledge and the high number of participating countries involved. This international collaboration is also important for pharmacovigilance activities, to enable the regulatory institutions such as the EMA to obtain fast and reliable information for population benefit-risk assessment. It also facilitates regulatory decision-making and assessing the public health impact of the use of medicines [13]. In addition, this compilation can promote networking between researchers and contribute to multidatabase studies which are of increasing interest for drug safety issues nowadays (ie, ESAC, TEDDY, GRIP).This work has provided not only an overview of the availability of drug utilisation data for the drug-adverse event pairs included in the PROTECT project, but also an update on the methodological framework for drug utilisation studies [10,14,15]. Finally, the access to the information and knowledge held by the groups are described. Researchers can use this information in pharmacoepidemiological studies to improve patient’s therapeutic management.

Our research has some limitations. First, some working groups could have been missed because of the difficulty in understanding some non-English language websites. Second, although we conducted a complete search, part of the results refers only to the drugs of interest for the PROTECT project. Third, the information about the data on medicines utilisation available for each working group has been extracted from their website or from the methods section in the published references, which is sometimes summarised. Finally, the update of the information has been difficult because the websites are not updated regularly.

## Conclusion

We assembled and updated information on European working groups in drug utilisation research and in the utilisation of five selected groups of drugs for the PROTECT project. A description of the main working groups and information on their data characteristics has been provided. This information should be of value for academic researchers, regulatory authorities, health authorities and pharmaceutical companies conducting and interpreting post-authorisation and safety studies. European and member states’ health authorities should encourage and support national research and European collaboration in this important field for public health.

## Abbreviations

ARITMO: Arrhythmogenic potential of drugs; ARPAC: Antibiotic resistance prevention and control; ARPEC: Antibiotic resistance and prescribing in European children; ATC: Anatomical therapeutic chemical; CNC: Cross national comparison; DDD: Defined daily dose; DG SANCO: Directorate-general for health and consumers; DRUID: Driving under the influence of drugs, alcohol and medicines; DU: Drug utilisation; DURQUIM: Drug utilisation research quality indicator meeting; EMA: European medicines agency; ENCePP: European network of centres for pharmacoepidemiology and pharmacovigilance; ESAC: European surveillance of antimicrobial consumption; ESEMeD: European study of the epidemiology of mental disorders; ESCMID-ESGAP: European society of clinical microbiology and infectious diseases- study group for antibiotic policies; EU: European union; EUROASPIRE: European action on secondary and primary prevention by intervention to reduce events; EuroDURG: European drug utilisation research group; EURO-MED-STAT: European medicines statistics; GRIP: Global research in paediatrics; HAPPY AUDIT: Health alliance for prudent, yield, and use of antimicrobial drugs in the treatment of respiratory tract infections; ISPE’S SIG-DUR: International society of pharmacoepidemiology special interest group of drug utilisation research; NorPEN: Nordic pharmacoepidemiological network; PROTECT: The pharmacoepidemiological research on outcomes of therapeutics by a European consortium; SIETES: Sistema de información esencial en terapéutica y salud; UK: United Kingdom; TEDDY: Task force in Europe for drug development for the young; TUPP/EUPoMMe: The users perspective project; WHO-DURG: World health organization drug utilisation research group.

## Competing interests

Sabaté M, Pacheco JF, Ballarín E, Ferrer P, Laporte J-R, Ibáñez L, Hasford J and Rottenkolber M declared that they do not have anything to disclose regarding funding or competing interests with respect to this manuscript. Petri H was employee of Roche until April 2012. Schoonen WM is an employee of Amgen Ltd. Fortuny J is an employee at Novartis Pharma AG. Costs related to their part in the research were carried by the respective company as in-kind contribution under the IMI JU scheme.

## Authors’ contributions

MS participated in the conception and design, data collection, data analysis, interpretation of data and writing the paper. LI participated in the conception and design, interpretation of data, and writing the paper. JP participated in the data analysis, and revising it critically for important intellectual content. EB participated in the conception and design, data collection, interpretation of data, data analysis, drafting the article and revising it critically for important intellectual content. PF participated in the conception and design, data collection, interpretation of data, and drafting the article, and revising it critically for important intellectual content. All other authors participated in the conception and design, interpretation of data, and revising it critically for important intellectual content. All authors read and approved the final manuscript.

## Supplementary Material

Additional file 1: Table S1 European working groups on general drug utilisation and on specific fields on drug utilisation. Click here for file
