# Supplementary material for: A compilation of research working groups on drug utilisation across Europe
Source: BMC Res Notes. 2014 Mar 13;7:143. doi: 10.1186/1756-0500-7-143 (PMC4008312; doi:10.1186/1756-0500-7-143)
Supplement: Additional file 1: Table S1 — European working groups on general drug utilisation and on specific fields on drug utilisation. [file 1756-0500-7-143-S1.docx]

Table S1. European working groups on general drug utilisation and on specific fields on drug utilisation

| **Name, Website^a^ and Funding** | **Information** |
| --- | --- |
| **Working groups promoting general DU research** | |
| **CNC**  *Cross National Comparison*  2008–ongoing <http://www.pharmacoepi.org/eurodurg/workgr/cross_national.cfm> | **Specific project**  **Objective:** Collect worldwide information on the state-of-the-art of national drug utilisation monitoring systems  **Data:** received by questionnaire to key informants and contact persons   - Poster presentation on drug **consumption data** (2000-2007)   ***Data source:*** dispensing or prescribing  ***Codification system:*** ATC  ***Units of measure:***DDD, € or $  ***Setting:*** outpatient  ***Period:* -**  ***Drugs of interest:*** Proton Pump Inhibitors (A02BC), Statines (C10AA), and clopidogrel (B01AC04) antibacterials (J01).  ***Covered population:* -** |
| **DURQUIM**  *Drug Utilisation Research Quality Indicator Meeting*  2004–ongoing  https://pharmacoepi.org/eurodurg/durquim.cfm  Funds: WHO/EuroDURG, RIZIV | **Expert meeting**  **Objective:**To analyse the patterns of drug use and to implement strategies for improving the prescribing and use of drugs, and constructing prescribing quality indicators.  **Data:** Power Point presentation of participating countries with the **available European databases** (administrative, prescription, reimbursing, health assurance, …).  ***Source, Codification system, Units of measure, Setting, Period, Drugs of interest, Covered population:*** no applicable. |
| **ENCePP**  *European Network of Centres for Pharmacoepidemiology and Pharmacovigilance*  2006–ongoing  <http://www.encepp.eu/>  Lead by the EMA | **Network**  **Objective:**To bring together the available expertise and research experience in the fields of pharmaco-epidemiology and pharmaco-vigilance scattered across Europe in a Network of Excellence.  **Data: Registry of EU data sources**(research and medical-care centers, healthcare databases, electronic registries and existing networks) in the field of pharmacoepidemiology and pharmacovigilance.  ***Source, Codification system, Units of measure, Setting, Period, Drugs of interest, Covered population:*** no applicable. |
| **EuroDURG**  *European Drug Utilisation Research Group*  1993–ongoing  EuroDURG bulletins <http://www.pharmacoepi.org/eurodurg>  *Funds:* WHO European Office | **Non-profit organization**  **Objective:** To provide an international forum for cooperation and communication to promote DU research, to work on: DU research methodology, cooperate with international and national drug regulatory authorities.  **Data:**  ***Source, Codification system, Units of measure, Setting, Period, Drugs of interest, Covered population:*** no applicable.  Working groups:  **DRUID**  **HAPPY AUDIT**  **TUPP**  **DURQUIM** |
| **EURO-MED-STAT**  2002–2007  <http://ec.europa.eu/eahc/projects/database.html?prjno=2003133>  *Funds:* European Commission | **Specific project**  **Objective:** To establish an **inventory of national medicines data sources** and a survey of available data, to assess data reliability and comparability between countries.  **Data:**   - List of medicines data sources (available on-line) - Competentauthorities of each country - list of licensed medicines per country. |
| **ISPE´s SIG-DUR**  *Special Interest Group of Drug Utilisation Research*  (2006–ongoing)  <http://www.pharmacoepi.org/resources/sigs_research.cfm> | **Network**  **Objective:** To create a forum for discussion and cooperation between drug utilization researchers.  **Data:** International ATC/DDD browser.  ***Source, Codification system, Units of measure, Setting, Period, Drugs of interest, Covered population:*** no applicable. |
|  | **MTI**  Methods for Testing Interventions. |
|  | **PCI**  Prescribing Quality Indicators. |
|  | **RHI**  Relationship with the Health Insurers |
| **NorPEN**  *Nordic Pharmacoepidemiological Network*  2008–ongoing  <http://www.nhv.se/customer/templates/InfoPage____1619.aspx?epslanguage=EN>  *Funds: NordForsk (Nordic research board operating under the Nordic council of Ministers)* | **Network**  **Objective:** To facilitate pharmacoepidemiological research initiatives promoting safer and more efficient drugs and drug use in a public health perspective.  **Data:** Database prescription drugs dispensed (for each country)  ***Source:***prescriptions  ***Codification system:*** ATC/DDD methodology  ***Units of measure:* -**  ***Setting:*** ambulatory care (in Denmark also in-patient)  ***Period:* -**  ***Drugs of interest:*** All drugs  ***Covered Population:*** 25 million people |
| **PIPERSKA GROUP**  2008–ongoing  <http://www.piperska.org/home> | **Network**  **Objective:** to ensure robust systems in place in Europe, to **enhance the rational use of drugs**, including new expensive drugs, to improve health.  **Data:**  ***Source, Codification system, Units of measure, Setting, Period, Covered population:*** no applicable.  ***Drugs of interest:*** New expensive drugs |
| **Working Groups focused on specific fields** | |
| **Antimicrobial drugs** | |
| **ARPAC**  *Antibiotic Resistence Prevention and Control*  2002–2005  <http://www.abdn.ac.uk/arpac>(not updated)  *Funds:* European Commission | **Specific project, network**  **Objective:** to develop strategies for control and prevention of antibiotic resistance in European hospitals.  **Data:**  ***Source:*** published data on antibiotic hospital use. Cross-sectional survey study (questionnaire survey).  ***Codification system:*** ATC  ***Units of measure:*** DDD/100 bed-days.  ***Setting:*** inpatient (170 hospitals)  ***Period:*** 2001 to 2005  ***Drugs of interest:*a**ntibiotic subgroups (4th ATC level)  ***Covered population:***- |
| **ARPEC** *Antibiotic Resistance and prescribing in European Children*  2010–ongoing  <http://www.arpec.sgul.ac.uk/>  *Funds:* DG SANCO of the European Union | **Specific project**  **Objective:** to improve the quality of antibiotic prescribing for children in Europe and to reduce the prevalence of antimicrobial resistance in bacterial infections in children.  **Data:** hospital point prevalence survey  ***Source:*** European community prescribing databases. Questionnaire to hospital pharmacists.  ***Codification system:*** ATC/DDD methodology  ***Units of measure:*** novel paediatric defined daily dose  ***Setting:*** in and out-patient  ***Period:* -**  ***Drugs of interest:* a**ntibiotic  ***Covered population:*-** |
| **ESGAP**  *ESCMID Study Group for Antibiotic Policies*  1998–ongoing  <http://www.escmid.org/research_projects/study_groups/antibiotic_policies/>  Official recognition from the ESCMID | **Study Group**  **Objective:** to improve antimicrobial prescribing policies and practices, to improve patient care and prevent/reduce the development of resistances  **Data:**  ***Source:***questionnaire distributed to hospital pharmacists  ***Codification system:*** ATC/DDD methodology  ***Units of measure:* ABC Calc:** to measure hospital antibiotic consumption in number of DDD/ 100 bed-days (available on the website).  ***Setting:*** hospital  ***Period:* -**  ***Drugs of interest:*** *a*ntibiotic  ***Covered population:*-** |
| **ESAC**  *European Surveillance of Antimicrobial Consumption*  2001–ongoing  <http://www.ecdc.europa.eu/en/activities/surveillance/esac-net/pages/index.aspx>*Funds:* DG SANCO of the European Union until 2006, European Centre for Disease Prevention and Control ECDC from 2007. | **Specific project, network**  **Objective:** to provide information on the consumption of antimicrobials and developing indicators and guidelines to help managing the risk of infections and resistances.  **Data:** interactive database available online  ***Source:*** distribution or reimbursement data (out-hospital)  Questionnaire to hospital pharmacist (in-hospital)  ***Codification system:*** ATC  ***Units of measure:*** DDD/1000 inhabitants/d  ***Setting:*** out and Inpatient and nursing homes  ***Period:*** annual reports by chemical group (ATC classification) since 2006–2008.  ***Drugs of interest:*** antiinfectives (J) ATC level 2 to 4.  ***Covered Population:*** population coverage: out-hospital <25-100%, In-hospital 9 countries sample data, the rest 85-100%. |
| **HAPPY AUDIT**  *Health Alliance for Prudent, Yield and Use of Antimicrobial Drugs in the treatment of respiratory tract infections.*  (2007-2010)  <http://www.happyaudit.org>  6^th^ FP | **Specific project**  **Objective:** to improve the antibiotics prescription in respiratory tract infections in primary health care in Europe through development of intervention program targeting general practitioners (GPs), parents of young children and healthy adults.  **Data:** report of the results (available on-line).  ***Source:*** according to the APO (*Audit Project Odense*): auditing GP. Sample of GP  ***Codification system: -***  ***Units of measure:*** antibiotic use in % by respiratory tract indication  ***Setting:*** outpatient  ***Period: -***  ***Drugs of interest:*** antibiotics  ***Covered population:* -** |
| **Cardiovascular disease** | |
| **EUROASPIRE**  *European Action on Secondary and Primary Prevention by Intervention to Reduce Events*  1994–ongoing  *Funds:* European Society of Cardiology registries.  <http://www.escardio.org/guidelines-surveys/ehs/prevention/Pages/ehs-on-prevention.aspx> | **Specific project**  **Objective:** to determine if cardiovascular risk factors are recorded in patients’ medical records, to measure the modifiable risk factors, to describe therapeutic management after hospitalization in patients with coronary hearth disease. To determine whether Joint European Guidelines on CV prevention are followed in every day clinical practice.  **Data:** published data  ***Source:*** review of medical records, and patient interviews.  ***Codification system: -***  ***Units of measure: -***  ***Setting:*** outpatient, inpatient (period 2006-2007)  ***Period:*** 1995-1996,1999-2000, 2006-2007.  ***Drugs of interest:*** antiplatelet, beta-blockers, ace inhibitors, calcium channel blockers, lipid-lowering drugs and anticoagulants by groups.  ***Covered Population:*** selected geographical area with a defined population and all hospitals serving this population |
| **ARITMO**  *Arrhythmogenic potential of drugs*  (Jan. 2010–Dec. 2012)  <http://www.aritmo-project.org>  *Funds:* FP7 | **Specific project , network**  **Objective:to analyse the arrhythmic potential of drugs.**  **Data:**  ***Source:*** review of literature and variety databases. Prospective case-control surveillance (planned).  ***Codification system:* -**  ***Units of measure:* -**  ***Setting:*** outpatient  ***Period:* -**  ***Drugs of interest:* antipsychotics, anti-infectives**(antibacterials, antimycotics and antivirals) and**H1-antihistaminics.**  ***Covered population:*-** |
| **Paedriatics** | |
| **TEDDY**  *Task Force in Europe for Drug Development for the Young*  (2005–2010)  <http://www.teddyoung.org/index>.php  *Funds:* FP6 | **Network, specific project**  **Objective:** to promote the availability of safe and effective medicines for children by integrating existing expertise and good practices.  **Data:** publications  ***Source:*** data sources containing information on medicines used in male/female children, out-patient data, prescription or drug dispensing data (Netherlands, Italy and UK).  ***Codification system: -***  ***Units of measure:*** prevalence drug of use by age and therapeutic level (ATC 2nd level)  ***Setting:*** outpatient  ***Period:*** 2000-2005  ***Drugs of interest:*** all drugs  ***Covered population:***2.9-100% |
| **Mental health/central nervous system affects** | |
| **ESEMeD**  *European Study of the Epidemiology of Mental Disorders*  (2001–2003)  Not website *Funds:* European Commission, WHO/World mental Health 2000 Initiative, GSK laboratories. | **Specific project**  **Objective:** to collect data by a cross-sectional survey, on prevalence, risk factors, health-related quality of life and use of services associated with common mental disorders.  **Data:** published data  ***Source:*** cross-sectional patients interview survey. Sample of the adult  ***Codification system: -***  ***Units of measure:*** presented in % of the total sample  ***Setting:*** outpatient  ***Period:*** 2001-2003  ***Drugs of interest:*** psychotropic drug  ***Covered population:* -** |
| **DRUID**  *Driving under the Influence of Drugs, Alcohol and Medicines*  (2006–2011)  <http://www.druid-project.eu/cln_031/nn_107542/Druid/EN/home/homepage__node.html?__nnn=true>  6^th^ FP | **Specific project**  **Objective:** to combat the scourge of drink-driving and find answers to the question of the use of drugs or medicines that affect people’s ability to drive safely.  **Data:** published data. Final report 1/8/2012. (Information is not currently available)  ***Source:*** data provided by national agencies, institutes of public health, insurance companies, community pharmacies, ministries of health.  ***Codification system:*** ATC/DDD methodology  ***Units of measure:* -**  ***Setting:*** outpatient  ***Period:*** 2000-2005.  ***Drugs of interest:*** anxiolytics, hypnotics, sedatives, antidepressants  ***Covered Population:*** 100% for 6 countries |
| **TUPP/EUPoMMe**  *European User’s Perspective on Mood-modifying Medicines*  (1997–ongoing)  http://www.pharmacoepi.org/eurodurg/workgr/tupp/index.htm (not updated) | **Specific project**  **Objective:** to frame a protocol for pan-European research on the user perspective on mood-modifying medicines.  **Data:**  ***Source:*** sample patient interview  ***Codification system:* -**  ***Units of measure:* -**  ***Setting: -***  ***Period: -***  ***Drugs of interest:*** mood-modifying medicines  ***Covered population:* -** |

^a^Last accessed for all websites 16/09/2013
